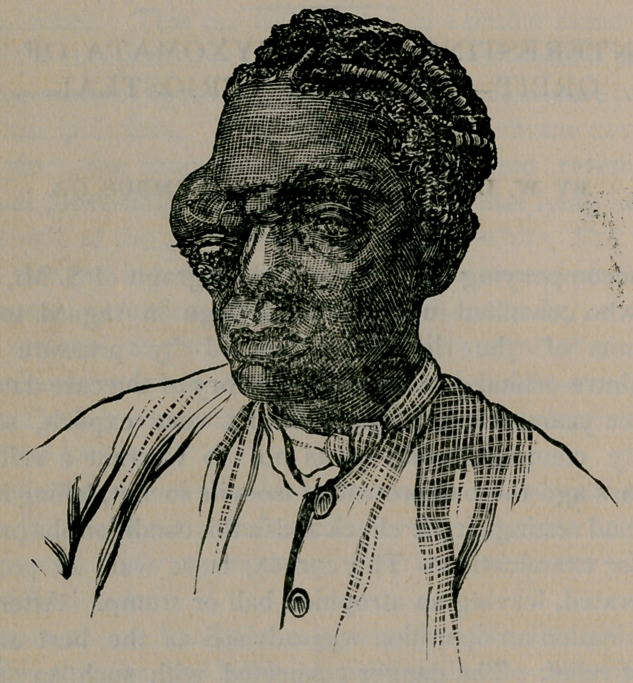# An Interesting Case—Myxomata of the Orbit—Probably Periosteal

**Published:** 1887-12

**Authors:** W. L. Bullard

**Affiliations:** Columbus, Ga.


### AN INTERESTING CASE—MYXOMATA OF THE ORBIT—PROBABLY PERIOSTEAL.

BY W. L. BULLARD, M. D., COLUMBUS, GA.

The accompanying cut is from a photograph of S. M., female, æt. 69, who consulted me a few weeks ago in regard to an exophthalmus of the right eye, caused by pressure behind from an intra-orbital growth. The history of the case dated back for twelve years, ten of which were not very explicit, so much ambiguity connected with it as not to warrant a reiteration. Two years ago the ball protruded from its socket, falling between the lids and resting on the cheek and in this condition she presented herself for examination. The cornea, from want of protection, had ulcerated, leaving an atrophied ball or stump. After a careful examination an operation was advised as the best and only means of relief. The danger connected with such an operation was made clear to the patient, yet she asked to have it done, saying that she preferred death to her condition. An anæsthetic, ether, was given. Drs. B anchard and Walker were present and kindly assisted in the operation. As anticipated, I soon saw that extirpation entire was impracticable, so proceeded as we would in orbital abscess, turning out quantities, three or four ounces of dark, thick gelatinous contents similar to that seen in ovarian cysts. On exploration with the finger more of the fluid was removed, and instead of finding the recti muscles, optic nerve, cellular tissue, etc., it entered a cavity extending as far back as the finger would reach, with a caving in, so to speak, of the roof, floor, outer and inner walls of the orbit. The cavity was washed out with carbolic acid solution, the incision brought together and held in apposition with sutures, a drainage-tube inserted, stump and curvated lids placed back in their normal position and bandaged.

As an antiseptic, corrosive sublimate solution 1 to 2,000

was used. There was a projection (one-half an inch or more) of new bone formation just below the superciliary ridge. The greater portion of this was removed. The incision (two inches long) healed by first intention, and patient recovered without any hyperexia, save on second day after operation, at which time the temperature ran up to 102°. It soon went down to normal and continued so through the after-treatment. Though confident that she will never make a complete recovery, yet she is now free from pain, is much better and stronger than she was prior to the operation, and, to use her own language, "her stomach has come back to her." The proptosis has receded, and the abnormality nothing compared to that before surgical interference. It was my intention, after removal of the growth, to replace stump, over which to fit an artificial eye, but absorption of the recti muscles, etc., rendered that impracticable.

## Figures and Tables

**Figure f1:**